# Evaluation of the mental health status of intensive care unit healthcare workers at the beginning of COVID-19 pandemic

**DOI:** 10.3389/fpubh.2024.1475107

**Published:** 2024-10-08

**Authors:** Ceren Meriç Özgündüz, Murat Bıçakçıoğlu, Ayse Sahin Tutak, Arman Özgündüz

**Affiliations:** ^1^Adıyaman University Research and Training Hospital, Psychiatry Department, Adıyaman, Türkiye; ^2^Adıyaman University Research and Training Hospital, Department of Anesthesiology and Reanimation, Adıyaman, Türkiye; ^3^Adıyaman University Faculty of Medicine, Department of Internal Diseases, Adıyaman, Türkiye; ^4^Adıyaman University Research and Training Hospital, Neurosurgery Department, Adıyaman, Türkiye

**Keywords:** COVID-19, mental health, depression, anxiety, stress, sleep quality, healthcare workers, intensive care unit

## Abstract

**Objective:**

During pandemic periods, mental health issues are highly prevalent, particularly among healthcare workers who are at a higher risk of developing psychiatric disorders. The aim of this study is to evaluate the mental health status of the intensive care unit (ICU) healthcare workers, who play a vital role in managing the COVID-19 pandemic, in terms of the quality of sleep, levels of depressive and anxiety symptoms, stress and to determine the factors that affect their mental health.

**Methods:**

The research was conducted in April 2020 and incorporated a total of 79 participants working in an university hospital ICUs in Turkey. Pittsburgh Sleep Quality Index (PSQI), Depression Anxiety Stress Scale 42 (DASS-42), Beck Depression Inventory (BDI), and Beck Anxiety Inventory (BAI) were applied.

**Results:**

Among the participants, 58 individuals comprising 73.4% of the cohort were working in the ICUs, managing patients infected with COVID-19. Those working in ICUs with COVID-19 patients had significantly higher DASS-S, BAI, and BDI scores. Doctors’ BDI scores were significantly lower compared to both nurses and other healthcare workers. Participants exhibiting COVID-19 symptoms manifested significantly higher BAI scores in compared to those without such symptoms.

**Conclusion:**

Healthcare workers involved in ICUs with COVID-19 patients were more significantly affected psychologically, doctors had lower depressive symptoms as compared to other healthcare workers. In addition, individuals with COVID-19 symptoms demonstrated significantly higher levels of anxiety. The findings of our study emphasize the significance of providing psychological support to healthcare workers throughout pandemics.

## 1 Introduction

Coronavirus disease 2019 (COVID-19), an infectious respiratory disease caused by a previously unidentified variant of coronavirus, known as severe acute respiratory syndrome coronavirus 2 (SARS-CoV-2), was first identified in December 2019 in the city of Wuhan, China and spread rapidly to the rest of the world (1). With a high transmittable rate, the virus can spread through direct human contact (2, 3). COVID-19 has the potential to manifest a diverse clinical spectrum of indications and symptoms, ranging from an asymptomatic state to the development of a Severe Acute Respiratory Syndrome (SARS) (4).

COVID-19 rapidly escalated into a global public health emergency and the World Health Organization (WHO) declared it as a pandemic on March 11, 2020. As of April 20, 2020, WHO reported 2,314,621 confirmed cases and 157,847 deaths worldwide due to COVID-19 (5). In Turkey, the number of confirmed cases was 90,980 and the number of deaths was 2,140 on the same date (6).

A pandemic is characterized as a disease or infectious factor that spreads globally or across national boundaries, affecting a substantial portion of the population (7). Research indicates that psychological conditions such as anxiety disorders, mood disorders, along with post-traumatic stress disorder (PTSD) have a tendency to escalate during pandemics (8). Given the mental health impact of pandemics, healthcare workers present a uniquely susceptible group owing to their heightened risk of infection, augmented work pressure, and dreadfulness of transmitting the virus to their families (9).

Indeed, healthcare workers’ mental health is associated with improved wellbeing, patient safety, and quality of care. The well-being of healthcare workers is crucial as it impacts the quality of care provided to patients. Especially nurses have been placed in unpredictable and high-risk situations during the COVID-19 pandemic, which has led to increased probabilities of physical, and mental distress, while impacting the quality and safety of the care they deliver (10–12).

Findings from research on preceding pandemics such as the SARS outbreak, showed that healthcare workers were at high risk for PTSD (13) and reported a high prevalence of anxiety and depression (14). Studies conducted throughout the COVID-19 pandemic also have reported a high incidence of depressive and anxiety symptoms, insomnia, and distress among healthcare workers, particularly women, nurses, and frontline healthcare workers entrusted with the diagnosis, treatment, and care of COVID-19 cases (11). An umbrella review has revealed that the COVID-19 pandemic has far-reaching effects on the mental health status of healthcare workers resulting in significant levels of anxiety, depression, PTSD, sleep disorders, and burnout (15). Healthcare workers, especially working in the emergency department, intensive care unit (ICU), and infectious diseases services have been recognized as a populace with a heightened risk of psychiatric disorders (16). These findings highlighted the need for increased awareness among hospital directors concerning the magnitude as well as the factors contributing to the psychological burden experienced by healthcare professionals (17).

Frontline healthcare workers undertaking the brunt of the responsibility, have undoubtedly been among the most severely affected groups, grappling with an amplified workload, encompassing the diagnosis and treatment of new infections, ascending stress levels, compromised or saturated healthcare systems, and an augmented risk of contagion (18). These incessant stressors could negatively impede their mental health and sleep quality. Due to their perpetual and close interactions with patients, not only face a heightened susceptibility to contracting infections, but they also experience high mental stress, which may result in disruptions in their sleep patterns (19). Timely prevention, accurate recognition/diagnosis, and treatment of anxiety and depression, paired with approaches designed to enhance sleep quality, are particularly imperative during exceedingly challenging periods, such as the COVID-19 pandemic (20).

The purpose of this study is to evaluate the mental health status of ICU healthcare workers, who play a vital role in managing the COVID-19 pandemic, in terms of the quality of sleep, levels of depressive and anxiety symptoms, and stress. Additionally, we aimed to reveal the factors that affect their mental health. By identifying psychologically high-risk groups and factors that may influence them during such periods, it would be possible to provide psychological support and measures to reduce insomnia, depression, anxiety, and distress.

## 2 Materials and methods

### 2.1 Procedure

This study was a cross-sectional study. Participants were given an informed consent form to read, and after deciding to participate voluntarily, they filled out a questionnaire regarding their sociodemographic data and COVID-19-related questions. Participants also completed the Pittsburg Sleep Quality Index (PSQI), Depression Anxiety Stress Scale 42 (DASS-42), Beck Depression Inventory (BDI), and Beck Anxiety Inventory (BAI) scales.

### 2.2 Sample

The study included healthcare workers who worked in the ICUs of an university hospital in Turkey between April 20–30, 2020. Hospital staff reported that 108 healthcare workers were being employed in the ICU during this time frame, but only 80 healthcare workers were able to be contacted due to the implementation of rotating overtime in public operations caused by the COVID-19 pandemic. Inclusion criteria for the study were being between the ages of 18–65, volunteering to participate, having at least primary school graduation and being literate. Exclusion criteria included not agreeing to participate in the study, being in a psychiatric treatment process, or declaring a chronic drug use that could affect anxiety, depression, stress levels, and insomnia. Only one healthcare worker declined to participate, leaving a sample of 79 participants.

### 2.3 Materials

Following the rules in the Scientific Advisory Board Study Guide for Health Institutions Working Guide and Infection Control Methods in the COVID-19 Pandemic, a face-to-face communication was held with the participants. The research was approved by the University Hospital Ethics Committee with Decree No: 2020/5-12.

#### 2.3.1 Questionnaire form of sociodemographic data and COVID-19 related questions

The researchers prepared a questionnaire form to assess the sociodemographic characteristics of the participants. The sociodemographic characteristics that were taken into consideration are age, sex, educational status, profession, the year in the profession, marital status, having a child, having a chronic illness, being in a psychiatric treatment process, chronic drug use, smoking and alcohol use. This form also included COVID-19 related questions regarding whether the participant worked in ICUs with COVID-19 patients, whether the participant had COVID-19 symptoms, whether the participant was residing with family members over 65 years of age and whether the participant underwent a COVID-19 diagnostic test.

#### 2.3.2 Pittsburg sleep quality index

The Pittsburg Sleep Quality Index (PSQI) is a self-report scale comprised of 19 questions that measure sleep quality. The scale assesses subjective sleep quality across 7 components and each item is rated on a score of 0–3. A higher total score, above 5, indicates poor sleep quality. The index was adapted to Turkish by Ağargün et al., and a validity and reliability study was conducted (21). In this study, Cronbach’s *α* value was calculated to assess the internal consistency of the scale, and it was 0.804. Permission was requested and obtained from the authors for the use of this scale.

#### 2.3.3 Depression anxiety stress scale 42

The Depression Anxiety Stress Scale 42 (DASS-42) is a self-report scale that evaluates depression, anxiety, and stress conditions dimensionally and categorically. The scale includes 42 items and requires a four-point Likert-type evaluation. In the original study, normal range values were established as 0–9 for depression, 0–7 for anxiety, and 0–14 for stress. The scale was adapted to Turkish by Bilgel et al., and a validity and reliability study was conducted (22). In this study, Cronbach’s *α* values were calculated to assess the internal consistency of the scale, and they were 0.92, 0.86, and 0.88 for depression, anxiety, and stress, respectively. These values revealed high internal consistency of the Turkish version of the DASS-42. Permission was requested and obtained from the authors for the use of this scale.

#### 2.3.4 Beck depression inventory

The Beck Depression Inventory (BDI) is a self-report scale designed to evaluate the risk, level, and change in the severity of depressive symptoms. The scale consists of 21 items rated on a three-point Likert-type evaluation. The total score ranges from 0 to 63, and a cut-off score of 17 was established in the validity and reliability study of the Turkish version of the scale. The BDI was developed by Beck (23) and was adapted to Turkish by Nesrin Hisli, who conducted a validity-reliability study (24). In this study, Cronbach’s *α* value was calculated to assess the internal consistency of the scale, and it was 0.80. Permission was requested and obtained from the author for the use of this scale.

#### 2.3.5 Beck anxiety inventory

The Beck Anxiety Inventory (BAI) is a self-report scale created by Beck et al. (25) to assess the anxiety symptoms. The scale includes 21 items rated on a three-point Likert-type evaluation. A validity and reliability study of the Turkish version of the scale was conducted by Ulusoy et al. (26). In this study, Cronbach’ s *α* value was computed for responses to the BAI and it was found 0.93. Permission was requested and obtained from the authors for the use of this scale.

### 2.4 Statistical analysis

The data were analyzed using SPSS 25.0, and descriptive statistics were presented as frequency, percentage, mean, and standard deviation values. The normal distribution of continuous variables was evaluated based on skewness and kurtosis levels. For paired comparison groups, T-Test and Mann–Whitney-U tests were employed, while Kruskal-Wallis tests were conducted for triple comparison groups. T-Test results for Independent Groups were reported as mean ± standard deviation values, while nonparametric tests included median, Q1, and Q3 values. Statistical significance was accepted at a level of *p* < 0.05.

## 3 Results

This study included a sample of 79 participants, consisting of 35 (44.3%) females and 44 (55.7%) males working in different ICUs. The participants’ ages were between 20 and 50 years, with a mean age of 33.01 ± 7.10. Among the participants, 58 individuals comprising 73.4% of the cohort were healthcare professionals working in the ICUs, managing patients infected with COVID-19. This subset consisted of 22 (27.8%) doctors, 43 (54.4%) nurses, and 14 (17.7%) other healthcare workers. Additionally, 21 (26.6%) participants had a family member with chronic disease, while 10 (12.7%) had a family member over the age of 65 at home. Moreover, 11 (13.9%) participants reported COVID-19 symptoms, and 16 (20.3%) underwent a COVID-19 diagnostic test, and all these results turned out to be negative (Table 1).

**Table 1:** Participants’ demographic characteristics and presence of family members with chronic diseases and over 65 years of age at home, having COVID-19 symptoms, and having a COVID-19 diagnostic test.

Variables		All data		COVID 19 ICU workers		Other ICU workers	
		*n*	%	*n*	%	*n*	%
Gender	Female	35	44.3	28	48.3	7	33.3
	Male	44	55.7	30	51.7	14	66.7
	Total	79	100	58	100	21	100
Marital status	Single	21	26.6	18	31	3	14.3
	Married	58	73.4	40	69	18	85.7
	Total	79	100	58	100	21	100
Profession	Doctor	22	27.8	14	24.1	8	38.1
	Nurse	43	54.4	34	58.6	9	42.9
	Other	14	17.7	10	17.2	4	19.0
	Total	79	100	58	100	21	100
Family member with chronic disease at home	No	58	73.4	43	74.1	15	71.4
	Yes	21	26.6	15	25.9	6	28.6
	Total	79	100	58	100	21	100
Family member over 65-year at home	No	69	87.3	50	86.2	19	90.5
	Yes	10	12.7	8	13.8	2	9.5
	Total	79	100	58	100	21	100
COVID-19 symptoms	No	68	86.1	49	84.5	19	90.5
	Yes	11	13.9	9	15.5	2	9.5
	Total	79	100	58	100	21	100
COVID-19 test	No	63	79.7	44	75.9	19	90.5
	Yes	16	20.3	14	24.1	2	9.5
	Total	79	100	58	100	21	100

According to the scales utilized in the research, it was discovered that 19 participants (24.1%) exhibited substandard sleep quality, 35 participants (44.3%) suffered from symptoms of depression, 51 participants (64.6%) reported symptoms of anxiety, and 44 (55.7%) experienced distress (Table 2).

**Table 2:** Distribution of participants by good/poor sleep quality, depression, anxiety, and high-stress rates.

Variables		*n*	%
Sleep quality (PSQI score > 5)	Good	60	75.9
	Poor	19	24.1
Depression (BDI score > 16)	No	44	55.7
	Yes	35	44.3
Anxiety (DASS-A score > 7)	No	28	35.4
	Yes	51	64.6
High stress (DASS-S score > 14)	No	35	44.3
	Yes	44	55.7

Furthermore, the participants were compared based on their workplace, specifically healthcare workers in ICUs with COVID-19 patients and those working in other ICUs, in terms of PSQI, DASS-S, BAI, and BDI scores. The results revealed no significant difference between the two groups in PSQI scores. However, the DASS-S, BAI, and BDI scores of participants working in ICUs with COVID-19 patients were significantly higher (Table 3).

**Table 3:** PSQI, DASS-S, BAI, and BDI mean scores of participants working in COVID-19 ICUs and participants working in other ICUs.

Variables	Participants	*n*	Mean	*S*	Confidence interval		*t*	*p*
					Minimum	Maximum		
PSQI	COVID-19 ICUs	58	9.00	3.95	−0.539	3.301	1.456	0.154
	Other ICUs	21	7.62	3.64				
DASS-S	COVID-19 ICUs	58	17.81	9.75	1.056	9.803	2.504	0.016
	Other ICUs	21	12.38	8.02				
BAI	COVID-19 ICUs	58	15.43	12.66	0.755	9.630	2.337	0.023
	Other ICUs	21	10.24	6.76				
BDI	COVID-19 ICUs	58	17.26	12.30	0.318	11.819	2.101	0.039
	Other ICUs	21	11.19	8.00				

When comparing doctors, nurses, and other healthcare workers across all participants, there was a significant difference in their BDI scores [χ^2^(2) = 11.72, *p* < 0.01]. Further analysis revealed that doctors’ BDI scores were significantly lower compared to both nurses and other healthcare workers. Additionally, nurses’ BDI scores were significantly lower compared to other healthcare workers (Table 4).

**Table 4:** Comparison of BDI scores for doctors, nurses, and other healthcare professionals.

Professions	*n*	Rn. Mean	Median	CRI (25–75%)	*Z*	*p*
Other HCP	14	25.75	23.0	13.75–30	−3.30	0.001
Doctors	22	13.89	9.50	2.75–15.50		
Nurses	43	36.57	13.0	8–23	−2.13	0.033
Doctors	22	26.02	9.50	2.75–15.50		
Other HCP	14	36.79	23.0	13.75–30	−2.02	0.043
Nurses	43	26.47	13.0	8–23		

Upon comparing the PSQI, DASS-S, BAI, and BDI scores of all participants, individuals experiencing COVID-19 symptoms demonstrated significantly higher BAI scores in comparison to their counterparts without COVID-19 symptoms (*p* < 0.01). When comparing participants working in ICUs with COVID-19 patients who had and had not experienced COVID-19 symptoms, those with symptoms had significantly higher BAI and DASS-S scores (*p* < 0.025, *p* < 0.01, respectively).

Furthermore, among participants working in ICUs with COVID-19 patients, those living with family members over 65 years of age were compared with those not living with such family members. It was found that those living with family members over 65 years of age had significantly higher BAI and DASS-S scores (*p* < 0.045, *p* < 0.016, respectively).

The correlation analysis revealed a negative correlation between age and DASS-S scores, as well as between years spent in the profession and BDI scores in all participants (*p* < 0.05) (Table 5).

**Table 5:** Correlations between participants’ age, years spent in the profession, PSQI, DASS-S, BAI, and BDI scores.

Variables	1	2	3	4	5	6
1-year	1					
2. Years spent in the profession	0.788^***^	1				
3. PSQI	−0.114	−0.258	1			
4. DASS-S	−0.305^*^	−0.246	0.539^***^	1		
5. BAI	−0.117	−0.087	0.438^***^	0.769^***^	1	
6. BDI	−0.116	−0.275^*^	0.319^*^	0.564^***^	0.607^***^	1

^*^p < 0.05, ^***^p < 0.00.

## 4 Discussion

This cross-sectional study was carried out on 79 healthcare professionals working in ICUs in an university hospital in Turkey. It is the initial study examining the mental state of ICU healthcare workers at the beginning of the COVID-19 in Turkey. Our study revealed that 19 participants (24.1%) exhibited substandard sleep quality, 35 participants (44.3%) suffered from symptoms of depression, 51 participants (64.6%) reported symptoms of anxiety, and 44 (55.7%) experienced distress, similar to the study conducted by Lai et al., involving 1,257 healthcare workers, psychiatric symptoms were detected to be elevated, for instance, 50.4% depressive symptoms, 44.6% anxiety symptoms, 34% insomnia, and 71.5% distress (11). Consistent with these findings, a meta-analysis of 38 studies, which was done during the COVID-19 pandemic, indicated that the pooled prevalence of mental health problems such as PTSD, anxiety, depression, and distress in healthcare professionals was 49, 40, 37, and 37%, respectively (27).

The study conducted by Lai et al., reported higher levels of psychological symptoms in healthcare workers involved in the diagnosis, treatment, and care of COVID-19 cases (11), as also discovered in our research. In consistent with these findings, the results of a systematic review of 29 studies demonstrated that the prevalence of stress, anxiety and depression within frontline healthcare workers caring for COVID-19 patients were high (28). In our study, frontline healthcare professionals working in ICUs with COVID-19 patients displayed higher levels of stress, anxiety, and depressive symptoms when compared to their colleagues working in other ICUs, with no significant difference according to sleep quality. This outcome could be attributed to the utilization of PSQI instead of the Insomnia Severity Index in our study, distinction regarding sampling, and ethnic characteristics. Alternatively, in a research involving 180 healthcare professionals in China, which examined the impact of social support on sleep quality, PSQI was used, and the participants had a mean PSQI score of 8.58 (29). Also, the participants of our study had a mean PSQI score of 8.63.

Lu et al. discovered that frontline healthcare workers operating in pulmonology, emergency, infectious diseases, and ICU services, closely serving COVID-19 patients, displayed higher anxiety levels compared to healthcare workers working in low-risk departments (30). In another research which investigated the factors affecting anxiety symptoms in healthcare workers in the course of the COVID-19 pandemic, individuals caring for COVID-19 patients showed significantly higher anxiety levels than individuals who did not (31). Again, a study conducted in Turkey discovered that working in a hospital where COVID-19 patients were admitted, increased the level of state anxiety (32). Due to COVID-19’s infectivity via person-to-person transmission, which is rapid and can be lethal, there is an intensified personal risk assessment in healthcare workers who are in contact with COVID-19 patients that increasing the risk of transmission (33, 34). During the initial stages of the COVID-19 pandemic, lack of experience and resources for combating the pandemic, coupled with extensive, high-pressure work environments, could have contributed to a heightened prevalence of anxiety and depression among frontline healthcare professionals (35). Moreover, the management of critically ill patients can impose immense pressure along with anxiety on the frontline healthcare workers (36).

Our study found that healthcare professionals working in ICUs had varying degrees of mental strain, and a lower tendency toward depressive symptoms was discovered among doctors when compared to nurses and other healthcare workers. As emphasized by Lai and colleagues, nurses exhibited higher incidence of depression, anxiety, insomnia, and stress in comparison to doctors, and this prevalence was associated with a greater number of women (90.8%) and individuals with lesser work experience (71.5%) in the group of nurses (11). A retrospective study also found higher levels of psychiatric disorders among female healthcare workers than their male counterparts (37). In our study, 65% of the nurses and 9% of the doctors were female. In line with these findings, the results of the systematic reviews which were conducted during the COVID-19 pandemic, showed that the prevalence of anxiety and depression in frontline healthcare workers was relatively high among women and nurses (35, 38). Another literature review conducted during the COVID-19 pandemic reported that nurses manifested more prominent depressive and anxiety symptoms in contrast to doctors (39). Moreover, it was ascertained that nurses were at increased risk of infection due to their proximity to patients, and were confronted with both physical as well as psychological hardships while fulfilling their responsibilities (40–42). As per another literature review published during the pandemic, nurses were observed to be in a psychologically susceptible group in contrast to doctors, and having less work experience was recognized as one of the potential risk factors (43). Due to staff shortages during the COVID-19 pandemic, many nurses were deployed to ICUs, even though they may not have had prior experience and training in caring for critically ill patients (44). The mean years of work experience for nurses in our study was 7.12, while that of doctors was 12.59.

Our findings were also in parallel with the findings of a meta-review which was about the mental health status and risk factors among global healthcare workers associated with the COVID-19 pandemic; it was stated that the most prevalent mental health problems identified in the review included anxiety, depression, and stress/PTSD and significant risk factors associated with the incidence of mental health issues include female gender, young age, low educational level, being a nurse, being a frontline health professional, experience, and country of residence (45). Another finding of this meta-review is similar to our finding unsurprisingly, as the rate of depression was higher among healthcare workers in contact with COVID-19 patients and those working in COVID-19 units, which is likely to be associated with increased interaction with dying or suffering patients and particularly these are the common situations seen in ICUs in which our study was conducted.

Our research conducted on healthcare workers working in ICUs with COVID-19 patients found that individuals residing with family members over 65 years of age experienced elevated levels of stress and anxiety symptoms as compared to those who did not. This is predominantly due to the heightened vulnerability of individuals beyond the age of 65 to COVID-19, which raises apprehensions for the well-being and safety of their cohabiting family members (46). In a research investigating the psychological impacts of SARS outbreak on healthcare professionals working in emergency departments, stress levels were affiliated not only with concerns for personal health but also with the health of their family members and others with regard to the dissemination of the virus (47). In a similar study examining the levels of despair and anxiety symptoms during the COVID-19 pandemic in Turkey, individuals cohabiting with family members classified as high-risk for COVID-19 presented conspicuously elevated levels of despair and anxiety symptoms in contrast to those not in such circumstances (32).

Our research also revealed that anxiety levels were higher in all participants with COVID-19 symptoms in comparison to those without symptoms. Moreover, among healthcare workers working in ICUs with COVID-19 patients, individuals exhibiting COVID-19 symptoms displayed higher levels of both anxiety symptoms and stress in comparison to those without symptoms. A study carried out throughout the COVID-19 pandemic on healthcare workers dealing with COVID-19 patients, revealed that being a suspected case of COVID-19 and residing in the Hubei region were linked to heightened levels of anxiety (31). It is noteworthy to consider that in our study, both managing COVID-19 patients and exhibiting COVID-19 symptoms are significant factors associated with heightened levels of not just anxiety but also stress. Together with these findings, it can be stated that the burden of stress among healthcare workers may be influenced by fear of getting infected or infecting family members in line with previous studies (48, 49).

Additionaly, according to our study, higher stress levels were associated with younger age, while higher depression levels were linked with less experience in the profession. A review published in 2020 identified less work experience and a younger age as probable risk factors for psychological distress among healthcare workers (43).

The limitations of our study primarily include its single-center design and sample consisting solely of healthcare workers in ICUs, which limits the generalizability of the outcomes. Additional limitations are the cross-sectional nature and the use of self-evaluation scales. However, a notable strength of our study is that it involved face-to-face communication with participants in compliance with the “Scientific Advisory Board Study Guide for Health Institutions Working Guide and Infection Control Methods in the COVID-19 Pandemic” suggested by the Turkish Ministry of Health, rather than being conducted online during the COVID-19 pandemic. Furthermore, our study’s ability to reach a substantial proportion (73%) of healthcare professionals working in ICUs in an university hospital under the COVID-19 pandemic working conditions represents another considerable strength.

## 5 Conclusion

The findings of our study can assist in quantifying the necessities of healthcare workers’ psychological support while delineating tiered and tailored interventions during pandemic periods. Imparting adequate psychological and social support to healthcare workers, especially working in frontline departments like ICUs during such periods holds significant importance, and hospital managements with mental health service units have a critical responsibility in this regard. Psychological status of the healthcare workers directly engaged with patients should be evaluated with particular attention to nurses, younger healthcare workers, and those with limited professional experience. Mental health support programs can help reduce stress, prevent burnout, and improve healthcare workers’ resilience, making them better equipped to care for their patients. Especially protecting the mental well-being of nurses is crucial to ensure the long-term capacity of the health workforce. Identifying and addressing the mental health status of nurses by providing necessary resources such as counseling services, peer support programs, and access to mental health professionals is essential.

As a result, priority should be given to the mental health of healthcare workers, particularly during times of crises, as their wellbeing is critical to the efficient functioning of the healthcare system. Understanding the factors that increase the risk of psychological problems among healthcare workers can aid in developing comprehensive strategies that prevent, manage, and minimize the exacerbation of mental health issues. For this reason, longitudinal research is crucial in comprehending the psychiatric consequences of the pandemic and prospective studies involving larger samples are necessary to decipher the role of psychosocial support and psychological interventions in dealing with these issues.

## Data Availability

The raw data supporting the conclusions of this article will be made available by the authors, without undue reservation.

## References

[1] Novel Coronavirus, Wuhan, China (2019). Information for Healthcare Professionals. Available online at: https://www.cdc.gov/coronavirus/2019-nCoV/hcp/index.html (Accessed April 20, 2020).

[2] Huang C Wang Y Li X Ren L Zhao J Hu Y. Clinical features of patients infected with 2019 novel coronavirus in Wuhan, China. Lancet. (2020) 395:497–506. doi: 10.1016/S0140-6736(20)30183-5, PMID: 31986264 PMC7159299

[3] Zhu N Zhang D Wang W Li X Yang B Song J. A novel coronavirus from patients with pneumonia in China, 2019. N Engl J Med. (2020) 382:727–33. doi: 10.1056/NEJMoa2001017, PMID: 31978945 PMC7092803

[4] da Silva FCT Neto MLR. Psychiatric symptomatology associated with depression, anxiety, distress, and insomnia in health professionals working in patients affected by COVID-19: A systematic review with meta-analysis. Prog Neuro-Psychopharmacol Biol Psychiatry. (2021) 104:110057. doi: 10.1016/j.pnpbp.2020.110057, PMID: 32777327 PMC7411383

[5] WHO (2020). Coronavirus disease (COVID-19) situation report-91. Available online at: https://www.who.int/docs/default-source/coronaviruse/situation-reports/20200420-sitrep-91-covid-19.pdf?sfvrsn=fcf0670b_10 (Accessed April 20, 2020).

[6] Republic of Turkey Ministry of Health (2020). Corona Table. Available online at: https://covid19.saglik.gov.tr/ (Accessed April 20, 2020).

[7] Porta MA. Dictionary of Epidemiology. New York, NY: Oxford University Press (2014).

[8] Taylor S. The Psychology of Pandemics. Newcastle upon Tyne, NE6 2PA, UK: Cambridge Scholars Publishing (2019).

[9] Xiang YT Yang Y Li W Zhang L Zhang Q Cheung T. Timely mental health care for the 2019 novel coronavirus outbreak is urgently needed. Lancet Psychiatry. (2020) 7:228–9. doi: 10.1016/S2215-0366(20)30046-8, PMID: 32032543 PMC7128153

[10] Huang L Lin G Tang L Yu L Zhou Z. Special attention to nurses’ protection during the COVID-19 epidemic. Crit Care. (2020) 24:120. doi: 10.1186/s13054-020-2841-7, PMID: 32220243 PMC7101882

[11] Lai J Ma S Wang Y Cai Z Hu J Wei N. Factors associated with mental health outcomes among health care workers exposed to coronavirus disease 2019. J Am Med Assoc Netw Open. (2020) 3:e203976. doi: 10.1001/jamanetworkopen.2020.3976 PMC7090843 32202646

[12] Rodríguez BO Sánchez TL. The psychosocial impact of COVID-19 on health care workers. Int Braz J Urol. (2020) 46:195–200. doi: 10.1590/S1677-5538.IBJU.2020.S124, PMID: 32618464 PMC7719993

[13] Wu P Fang Y Guan Z Fan B Kong J Yao Z. The psychological impact of the SARS epidemic on hospital employees in China: exposure, risk perception, and altruistic acceptance of risk. Can J Psychiatr. (2009) 54:302–11. doi: 10.1177/070674370905400504 PMC3780353 19497162

[14] Chong MY Wang WC Hsieh WC Lee CY Chiu NM Yeh WC. Psychological impact of severe acute respiratory syndrome on health workers in a tertiary hospital. Br J Psychiatry. (2004) 185:127–33. doi: 10.1192/bjp.185.2.127 15286063

[15] Chirico F Ferrari G Nucera G Szarpak L Crescenzo P Ilesanmi O. Prevalence of anxiety, depression, burnout syndrome, and mental health disorders among healthcare workers during the COVID-19 pandemic: a rapid umbrella review of systematic reviews. J Health Soc Sci. (2021) 6:209–20. doi: 10.19204/2021/prvl7

[16] Naushad VA Bierens JJ Nishan KP Firjeeth CP Mohammad OH Maliyakkal AM. A systematic review of the impact of disaster on the mental health of medical responders. Prehosp Disaster Med. (2019) 34:632–43. doi: 10.1017/S1049023X19004874, PMID: 31625487

[17] Tam CW Pang EP Lam LC Chiu HF. Severe acute respiratory syndrome (SARS) in Hong Kong in 2003: stress and psychological impact among frontline healthcare workers. Psychol Med. (2004) 34:1197–204. doi: 10.1017/S0033291704002247, PMID: 15697046

[18] Yang Y Li W Zhang Q Zhang L Cheung T Xiang YT. Mental health services for older adults in China during the COVID-19 outbreak. Lancet Psychiatry. (2020) 7:e19. doi: 10.1016/S2215-0366(20)30079-1, PMID: 32085843 PMC7128970

[19] Qi J Xu J Li BZ Huang JS Yang Y Zhang ZT. The evaluation of sleep disturbances for Chinese frontline medical workers under the outbreak of COVID-19. Sleep Med. (2020) 72:1–4. doi: 10.1016/j.sleep.2020.05.023 32502844 PMC7244440

[20] Huang Y Zhao N. Generalized anxiety disorder, depressive symptoms and sleep quality during COVID-19 outbreak in China: a web-based cross-sectional survey. J Psychiatr Res. (2020) 288:112954. doi: 10.1016/j.psychres.2020.112954, PMID: 32325383 PMC7152913

[21] Ağargün MY Kara H Anlar Ö. Pittsburgh Uyku Kalitesi İndeksi’ nin Geçerliği ve Güvenirliği. Turk Psikiyatri Derg. (1996) 7:107–15.

[22] Bilgel N Bayram N. Depresyon Anksiyete Stres Ölçeğinin (DASS-42) Türkçeye Uyarlanmış Şeklinin Psikometrik Özellikleri. Nöropsik Arşivi. (2010) 47:118–26.

[23] Beck AT. An inventory for measuring depression. Arch Gen Psychiatry. (1961) 4:561–71. doi: 10.1001/archpsyc.1961.01710120031004 13688369

[24] Hisli N. Beck Depresyon Envanterinin Üniversite Öğrencileri için Geçerliği. Güvenirliği. Psikol Derg. (1989) 7:3–13.

[25] Beck AT Epstein N Brown G Steer RA. An inventory for measuring clinical anxiety: psychometric properties. J Consult Clin Psychol. (1988) 56:893–7. doi: 10.1037/0022-006X.56.6.893 3204199

[26] Ulusoy M Şahin N Erkmen H. Turkish version of the Beck anxiety inventory: psychometric properties. J Cogn Psychother. (1998) 12:28–35.

[27] Saragih ID Tonapa SI Saragih IS Advani S Batubara SO Suarilah I. Global prevalence of mental health problems among healthcare workers during the covid-19 pandemic: a systematic review and meta-analysis. Int J Nurs Stud. (2021) 121:104002. doi: 10.1016/j.ijnurstu.2021.104002, PMID: 34271460 PMC9701545

[28] Salari N Khazaie H Hosseinian-Far A Khaledi-Paveh B Kazeminia M Mohammadi M. The prevalence of stress, anxiety and depression within front-line healthcare workers caring for COVID-19 patients: a systematic review and meta-regression. Hum Resour Health. (2020) 18:100. doi: 10.1186/s12960-020-00544-1, PMID: 33334335 PMC7745176

[29] Xiao H Zhang Y Kong D Li S Yang N. The effects of social support on sleep quality of medical staff treating patients with coronavirus disease 2019 (COVID-19) in January and February 2020 in China. Med Sci Monit. (2020) 26:e923549. doi: 10.12659/MSM.923549, PMID: 32132521 PMC7075079

[30] Lu W Wang H Lin Y Li L. Psychological status of medical workforce during the COVID-19 pandemic: A cross-sectional study. J Psychiatr Res. (2020) 288:112936. doi: 10.1016/j.psychres.2020.112936, PMID: 32276196 PMC7195354

[31] Liu CY Yang YZ Zhang XM Xu X Dou QL Zhang WW. The prevalence and influencing factors in anxiety in medical workers fighting COVID-19 in China: a cross-sectional survey. Epidemiol Infect. (2020) 148:e98. doi: 10.1017/S0950268820001107 32430088 PMC7251286

[32] Hacimusalar Y Kahve AC Yasar AB Aydin MS. Anxiety and hopelessness levels in COVID-19 pandemic: A comparative study of healthcare professionals and other community sample in Turkey. J Psychiatr Res. (2020) 129:181–8. doi: 10.1016/j.jpsychires.2020.07.024, PMID: 32758711 PMC7372275

[33] Li Q Guan X Wu P Wang X Zhou L Tong Y. Early transmission dynamics in Wuhan, China, of novel coronavirus-infected pneumonia. N Engl J Med. (2020) 382:1199–207. doi: 10.1056/NEJMoa2001316 31995857 PMC7121484

[34] Wang W Tang J Wei F. Updated understanding of the outbreak of 2019 novel coronavirus (2019-nCoV) in Wuhan, China. J Med Virol. (2020) 92:441–7. doi: 10.1002/jmv.25689, PMID: 31994742 PMC7167192

[35] Chen Y Wang J Geng Y Fang Z Zhu L Chen Y. Meta-analysis of the prevalence of anxiety and depression among frontline healthcare workers during the COVID-19 pandemic. Front Public Health. (2022) 10:984630. doi: 10.3389/fpubh.2022.984630, PMID: 36176525 PMC9513468

[36] Chan-Yeung M. Severe acute respiratory syndrome (SARS) and healthcare workers. Int J Occup Environ Health. (2004) 10:421–7. doi: 10.1179/oeh.2004.10.4.421 15702757

[37] Kim MS Kim T Lee D Yook JH Hong YC Lee SY. Mental disorders among workers in the healthcare industry: 2014 national health insurance data. Ann Occup Environ Med. (2018) 30:31. doi: 10.1186/s40557-018-0244-x, PMID: 29755753 PMC5934846

[38] Pappa S Ntella V Giannakas T Giannakoulis VG Papoutsi E Katsaounou P. Prevalence of depression, anxiety, and insomnia among healthcare workers during the COVID-19 pandemic: a systematic review and meta-analysis. Brain Behav Immun. (2020) 88:901–7. doi: 10.1016/j.bbi.2020.05.026, PMID: 32437915 PMC7206431

[39] Spoorthy MS Pratapa SK Mahant S. Mental health problems faced by healthcare workers due to the COVID-19 pandemic—a review. Asian J Psychiatr. (2020) 51:102119. doi: 10.1016/j.ajp.2020.102119, PMID: 32339895 PMC7175897

[40] Chan S. Nurses fighting against severe acute respiratory syndrome (SARS) in Hong Kong. J Nurs Scholarsh. (2003) 35:209. doi: 10.1111/j.1547-5069.2003.00209.x, PMID: 14562486

[41] Shih FJ Gau ML Kao CC Yang CY Lin YS Liao YC. Dying and caring on the edge: Taiwan’s surviving nurses’ reflections on taking care of patients with severe acute respiratory syndrome. Appl Nurs Res. (2007) 20:171–80. doi: 10.1016/j.apnr.2006.08.007, PMID: 17996803 PMC7127079

[42] Tzeng HM. Fighting the SARS epidemic in Taiwan: a nursing perspective. J Nurs Adm. (2003) 33:565–7. doi: 10.1097/00005110-200311000-00005 14608214

[43] Kisely S Warren N McMahon L Dalais C Henry I Siskind D. Occurrence, prevention, and management of the psychological effects of emerging virus outbreaks on healthcare workers: rapid review and meta-analysis. Br Med J. (2020) 369:m1642. doi: 10.1136/bmj.m1642, PMID: 32371466 PMC7199468

[44] Stayt LC Merriman C Bench S Price A Vollam S Walthall H. Doing the best we can': registered Nurses' experiences and perceptions of patient safety in intensive care during COVID-19. J Adv Nurs. (2022) 78:3371–84. doi: 10.1111/jan.15419, PMID: 35986583 PMC9538018

[45] Chutiyami M Cheong AMY Salihu D Bello UM Ndwiga D Maharaj R. COVID-19 pandemic and overall mental health of healthcare professionals globally: A Meta-review of systematic reviews. Front Psychol. (2022) 12:804525. doi: 10.3389/fpsyt.2021.804525 PMC8801501 35111089

[46] Perrotta F Corbi G Mazzeo G Boccia M Aronne L D'Agnano V. COVID-19 and the elderly: insights into pathogenesis and clinical decision-making. Aging Clin Exp Res. (2020) 32:1599–608. doi: 10.1007/s40520-020-01631-y, PMID: 32557332 PMC7298699

[47] Wong TW Yau JK Chan CL. The psychological impact of severe acute respiratory syndrome outbreak on healthcare workers in emergency departments and how they cope. Eur J Emerg Med. (2005) 12:13–8. doi: 10.1097/00063110-200502000-00005, PMID: 15674079

[48] Labrague LJ De los Santos JAA. COVID-19 anxiety among front-line nurses: predictive role of organisational support, personal resilience and social support. J Nurs Manag. (2020) 28:1653–61. doi: 10.1111/jonm.13121, PMID: 32770780 PMC7436313

[49] Yue C Liu C Wang J Zhang M Wu H Li C. Association between social support and anxiety among pregnant women in the third trimester during the coronavirus disease 2019 (COVID-19) epidemic in Qingdao, China: the mediating effect of risk perception. Int J Soc Psychiatry. (2021) 67:120–7. doi: 10.1177/0020764020941567, PMID: 32643510 PMC7348553

[50] Ozgunduz C Bıçakçıoğlu M Tutak A Ozgunduz A. P.0641 mental health status of the intensive care unit healthcare workers in an university hospital at the beginning of COVID-19 pandemic. Eur Neuropsychopharmacol. (2021) 53:S471–2. doi: 10.1016/j.euroneuro.2021.10.605

